# Healthy vaccinee effect in the evaluation of updated COVID-19 vaccines in elderly populations

**DOI:** 10.1038/s41467-026-76312-x

**Published:** 2026-08-08

**Authors:** Johan Lyth, Armin Spreco, Jorma Hinkula, Olle Björneld, Olle Eriksson, Dennis Nordvall, Örjan Dahlström, Elin A. Gursky, Thomas Schön, Toomas Timpka

**Affiliations:** 1 Department of Health, Medicine and Caring Sciences, Linköping University, Linköping University, Linköping, Sweden; 2 Regional Executive Office, Östergötland County Council, Linköping, Sweden; Department of Medical and Health Sciences, Linköping University, Linköping, Sweden; 3 Department of Biomedical and Clinical Sciences, Linköping University, Linköping, Sweden; 4 Data Intensive Sciences and Applications (DISA-IDP), Department of Computer science and Media technology (CM), Faculty of Technology, Linnaeus University, Kalmar, Sweden; 5 Data and Analysis, IT Division, Region Kalmar County, Kalmar, Sweden; 6 Qulturum Development Department, Region Jönköping County, Jönköping, Sweden; 7 Department of Behavioural Sciences and Learning, Linköping University, Linköping, Sweden; 8 College of Osteopathic Medicine, William Carey University, Hattiesburg, MS USA; 9 Department of Infectious Diseases, Kalmar County Hospital, Linköping University, Kalmar, Sweden

**Keywords:** Epidemiology, Outcomes research, Health policy

## Abstract

Established determinants of health in the elderly help guide routines for indicated vaccine administration, while unmeasured frailty may limit vaccine access. We evaluate the performance of the 2024-2025 COVID-19 vaccine adapted to the Omicron JN.1 lineage in a Swedish population aged ≥65 years (*N* = 245 696). Vaccine effectiveness (VE) on COVID-19-related hospitalization and a negative control outcome (NCO; all-cause mortality) are assessed in various cohorts between October 1, 2024 to March 31, 2025. The VE was 75% (95% CI 70%–79%) overall, and 84% (95% CI 80%–87%) and 65% (95% CI 34%–82%) in individuals with and without vaccination with the prior updated COVID-19 vaccine in 2023-2024, respectively. The NCO in individuals exposed to the study vaccine in 2024-2025 was half of that seen in those not exposed to the vaccine (HR 0.43 [95% CI 0.40–0.45]). Removing individuals hospitalized with COVID-19 from this population did not change the difference in NCO (HR 0.43 [95% CI 0.41-0.46]). These findings suggest the presence of selection effects arising from under-provision of health services to elderly individuals with frailty. The healthy vaccinee effect should be considered in observational studies of the effectiveness of updated COVID-19 vaccines in elderly populations.

## Abstract (web summary)

This Swedish population study reveals that healthy vaccinee bias inflates estimates of the effectiveness of updated COVID-19 vaccines in adults aged 65 years or older, highlighting the need to account for frailty and overall health status.

## Introduction

Post-pandemic revisions to COVID-19 vaccine recommendations often prioritize the elderly and patients with co-morbidities to receive an updated COVID-19 vaccine^1^. In August 2024, the FDA approved mRNA COVID-19 vaccines adapted to the Omicron JN.1 lineage by Moderna and Pfizer-BioNTech (specifically the KP.2 strain)^2^. The JN.1 lineage had more than 30 mutations in the spike protein compared to the Omicron XBB.1.5 lineage selected for vaccine updates in 2023. Reports from evaluations of the 2024-2025 mRNA COVID-19 vaccines have shown protection against COVID-19 morbidity, albeit at various levels. An early study using test-negative design noted 75% vaccine effectiveness (VE) in preventing COVID-19 hospitalization with the updated BNT162b2 vaccine adapted to the Omicron JN.1 lineage in a US population of army veterans aged ≥ 65 years^3^. The protection was even higher among individuals having received the 2023 vaccine adapted to the Omicron XBB.1.5 lineage. A study using a similar design conducted in the VISION and IVY networks noted lower VE (45% in immunocompetent and 40% in immunocompromised elderly individuals)^4^, while a parallel Danish cohort study in a population having received the 2023–24 Omicron XBB.1.5-adapted vaccine reported 70% VE in preventing COVID-19 hospitalization^5^.

There may be several reasons for variations in VE estimates in populations aged ≥65 years. One potential is the influence of a healthy vaccinee effect, i.e., that severely ill or otherwise disadvantaged individuals are less likely to be vaccinated. Estimates of VE from observational studies can be affected by various forms of bias, e.g., indication and healthy vaccinee effects^6^. The indication effect arises when individuals with underlying health conditions are enabled to receive vaccination, while the healthy vaccinee effect occurs when healthier persons are more likely to receive the vaccine than their less healthy peers. Both effects have been discussed in studies of COVID-19 vaccine effectiveness in general populations^7–11^. In evaluations of influenza vaccine effectiveness, the healthy vaccinee effect has been shown to particularly affect the elderly^12,13^.

In October 2024, residents ≥65 years of age in the Southeast region of Sweden (total population 1.2 million), were offered vaccination with the updated BNT162b2 COVID-19 vaccine^14^. We examined how healthy vaccinee status impacted estimates of VE in this setting between October 1, 2024, and March 31, 2025. We first estimated the effectiveness of the updated vaccine on COVID-19-related hospitalization in the total population. We then investigated the corresponding VE in subpopulations assumed to be differently affected by a potential healthy vaccinee effect, i.e. in individuals having and not having received the Omicron XBB.1.5 lineage vaccine in the previous year. We also investigated the negative control outcome (NCO), all-cause mortality in individuals exposed and not exposed to vaccination during the study period. An NCO is a factor acting as a surrogate outcome that is not causally affected by the exposure of interest^15^. Short-term all-cause mortality has been proposed as a suitable NCO for vaccine-effectiveness studies among the elderly^12^.

**Fig. 1:**
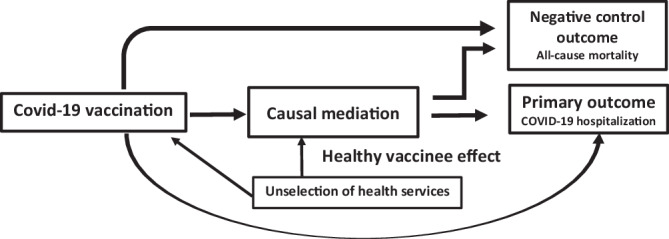
Hypothesized model of healthy vaccinee effect in evaluations of updated COVID-19 vaccines in elderly populations. Direct and indirect associations between exposure to COVID-19 vaccination and the primary outcome, COVID-19 hospitalization, and the negative control outcome, all-cause mortality, are displayed. The healthy vaccinee effect is assumed to be mediated by the selective under-provision of health services.

We hypothesized the healthy vaccinee effect to be associated with selective under-provision of preventive interventions such as vaccinations in elderly populations (Fig. 1). Potential healthy vaccinee effects were anticipated to show as VE differences between the subpopulations having and not having received the prior updated COVID-19 vaccine, and in the incidence of the NCO in those exposed and not exposed to the updated vaccine in the study year. To estimate the size of a potential healthy vaccinee effect, we simulated a counterfactual scenario in which causal mediators of vaccine effect on the study outcome, COVID-19 hospitalization, were eliminated. A corresponding scenario was used to validate the NCO.

## Results

The study population included 245,696 individuals aged ≥65 years. Uptake of the updated vaccine in 2024 was 68.0% (n = 166 975) (Table 1); most individuals (95.7%) were vaccinated in October and November (median October 2024). When compared to individuals with no completed basic COVID-19 vaccination, individuals not vaccinated with the Omicron XBB.1.5-adapted vaccine in 2023 but with a history of basic COVID-19 vaccination were less likely to be vaccinated in 2024 (adjusted OR (aOR) 4.8 [95% CI 4.4–5.1]) than those vaccinated with the updated vaccine in 2023 (aOR 120 [95% CI 112–129]) (Table 2). Also, individuals born outside Sweden were less likely to be vaccinated (aOR 0.45 [95% CI 0.43–0.46]). Considering comorbidities, the largest deviance in vaccination rates showed as reduced uptakes in individuals diagnosed with metastatic cancer (aOR 0.85 [95% CI 0.77–0.93]) and hemiplegia/paraplegia (aOR 0.80 [95% CI 0.73–0.89]) (Table 3). Immunocompromised individuals were more likely to be vaccinated than those immunocompetent (aOR 1.08 [95% CI 1.01–1.16]).

**Table 1:** Exposure status and outcomes of interest in subgroups of the total population aged ≥65 years in Southeast Sweden (*n* = 245,696)

	Vaccination uptake No. (%)			COVID-19 hospitalization No. (Daily rate per 100 000 persons)					All-cause mortality No. (Daily rate per 100 000 persons)		
	Updated COVID-19 vaccine				Updated COVID-19 vaccine			Updated COVID-19 vaccine			
	Yes	No	Total (100%)		Yes	No	Total	Yes		No	Total
**Total**	166 975 (68.0)	78 721 (32.0)	245 696		224 (0.887)	295 (2.110)	519 (1.323)	2 607 (10.31)		2 215 (15.81)	4 822 (12.27)
**Age group (yrs.)**											
65–69	32 842 (55.4)	26 430 (44.6)	59 272		11 (0.225)	27 (0.567)	38 (0.393)	83 (1.694)		178 (3.736)	261 (2.700)
70–74	37 498 (66.4)	19 011 (33.6)	56 509		22 (0.389)	36 (1.055)	58 (0.640)	208 (3.681)		251 (7.350)	459 (5.063)
75–79	41 094 (72.7)	15 403 (27.3)	56 497		44 (0.706)	69 (2.518)	113 (1.260)	374 (5.999)		382 (13.90)	756 (8.417)
80–84	29 779 (75.9)	9 477 (24.1)	39 256		59 (1.290)	61 (3.676)	120 (1.926)	510 (11.14)		456 (27.38)	966 (15.47)
85+	25 762 (75.4)	8 400 (24.6)	34 162		88 (2.252)	102 (7.256)	190 (3.576)	1 432 (36.58)		948 (66.90)	2 380 (44.64)
**Sex**											
Female	88 774 (68.5)	40 842 (31.5)	129 616		99 (0.736)	138 (1.901)	237 (1.144)	1 403 (10.42)		1 127 (15.49)	2 530 (12.20)
Male	78 201 (67.4)	37 879 (32.6)	116 080		125 (1.059)	157 (2.337)	282 (1.522)	1 204 (10.19)		1 088 (16.15)	2 292 (12.35)
**Immunosuppression**^**a**^											
Immunocompetent	133 648 (66.8)	66 508 (33.2)	200 156		138 (0.682)	188 (1.584)	326 (1.015)	1 660 (8.195)		1 385 (11.64)	3 045 (9.471)
Immunocompromised	33 327 (73.2)	12 213 (26.8)	45 540		86 (1.715)	107 (5.082)	193 (2.711)	947 (18.86)		830 (39.22)	1 777 (24.90)
**Country of birth**											
Sweden	156 765 (71.5)	62 400 (28.5)	219 165		215 (0.907)	262 (2.370)	477 (1.372)	2 428 (10.23)		1 913 (17.26)	4 341 (12.47)
Other	10 210 (38.5)	16 321 (61.5)	26 531		9 (0.583)	33 (1.130)	42 (0.941)	179 (11.59)		302 (10.32)	481 (10.76)
**COVID-19 vaccination**											
XBB.1.5-adjusted 2023	155 917 (87.6)	22 055 (12.4)	177 972		213 (0.901)	162 (4.232)	375 (1.365)	2 440 (10.31)		1 192 (30.98)	3 632 (13.20)
Earlier doses ( ≥ 2) only	10 230 (20.0)	40 840 (80.0)	51 070		10 (0.669)	102 (1.394)	112 (1.271)	158 (10.57)		711 (9.705)	869 (9.851)

^a^ Diagnoses used for classification of immunosuppression status: B20*, B21*, B22*, B23*, C*, D279, D610, D612, D619, D700, D80*, D810, D811, D812, D814, D815, D816, D817, D818, D819, D82*, D83*, D84*, D86*, D890, D891, D893, D894, D898, D899, E851, E852, E853, E854, E858, E859, G35*, J679, K703, K704, K72*, K743, K744, K745, K746, L930, L932, L94*, M05*, M06*, M07*, M08*, M30*, M313, M315, M32*, M33*, M34*, M353, M359, M460, M461, M468, M469, N04*, O987, R180, T860, T861, T862, T863, T864, T865, Z21*, Z482, Z510, Z511, Z94*.

**Table 2:** Immunosuppression and COVID-19 vaccination history in vaccinated and unvaccinated individuals aged ≥65 years in the Southeast region of Sweden, October 1, 2024 to March 31, 2025 (n = 245.696)

	Vaccination uptake No. (%) Updated COVID-19 vaccine		Unadjusted OR (95% CI)	*P*	Adjusted^a^ OR (95% CI)	*P*
	Yes	No				
**Total**	166 975 (100.0)	78 721 (100.0)				
**Immunosuppression**^b^						
Immunocompetent (Reference)	133 648 (80.0)	66 508 (84.5)				
Immunocompromised	33 327 (20.0)	12 213 (15.5)	1.36 (1.33 – 1.39)	<0.001	1.08 (1.01 – 1.16)	0.02
**COVID-19 vaccination history**						
Incomplete (0-1 doses) (Reference)	828 (0.5)	15 826 (20.1)				
Basic ( ≥ 2 doses)	10 230 (6.1)	40 840 (51.9)	4.8 (4.5 – 5.2)	<0.001	4.8 (4.4 – 5.1)	<0.001
XBB 1.5-adapted vaccine 2023	155 917 (93.4)	22 055 (28.0)	135 (126 – 145)	<0.001	120 (112 – 129)	<0.001

*OR* Odds ratio, 95% *CI* 95% Confidence intervals.

^a^Adjusted by diagnoses in the Charlson Comorbidity index^14^ past two years, age, sex and previous Covid hospitalization.

^b^Diagnoses used for classification as immunocompromised: B20*, B21*, B22*, B23*, C*, D279, D610, D612, D619, D700, D80*, D810, D811, D812, D814, D815, D816, D817, D818, D819, D82*, D83*, D84*, D86*, D890, D891, D893, D894, D898, D899, E851, E852, E853, E854, E858, E859, G35*, J679, K703, K704, K72*, K743, K744, K745, K746, L930, L932, L94*, M05*, M06*, M07*, M08*, M30*, M313, M315, M32*, M33*, M34*, M353, M359, M460, M461, M468, M469, N04*, O987, R180, T860, T861, T862, T863, T864, T865, Z21*, Z482, Z510, Z511, Z94*.

**Table 3:** Co-morbidity in disorders included in the Charlson Comorbidity Index in vaccinated and unvaccinated individuals aged ≥65 years in the Southeast region of Sweden, October 1, 2024 to March 31, 2025 (n = 245.696)

	Vaccination uptake No. (%) Updated COVID-19 vaccine		Unadjusted OR (95% CI)	*P*	Adjusted^a^ OR (95% CI)	*P*
	Yes	No				
**Total**	166 975 (100.0)	78 721 (100.0)				
**Comorbidity**^b^						
Charlson index (mean (sd).)	1.1 (1.7)	0.9 (1.6)				
Myocardial infarction	12 539 (7.5)	5 237 (6.7)	1.14 (1.10 – 1.18)	< 0.001	0.99 (0.94 – 1.04)	0.62
Congestive heart failure	17 257 (10.3)	6 339 (8.1)	1.32 (1.28 – 1.36)	< 0.001	1.00 (0.95 – 1.04)	0.86
Peripheral vascular disease	8 188 (4.9)	3 294 (4.2)	1.18 (1.13 – 1.23)	< 0.001	0.94 (0.89 – 1.00)	0.04
Cerebrovascular disease:	15 689 (9.4)	6 069 (7.7)	1.24 (1.20 – 1.28)	< 0.001	1.02 (0.97 – 1.06)	0.45
Dementia	8 431 (5.0)	2 666 (3.4)	1.52 (1.45 – 1.59)	< 0.001	0.97 (0.92 – 1.03)	0.40
Chronic pulmonary disease	24 403 (14.6)	9 448 (12.0)	1.25 (1.22 – 1.29)	< 0.001	1.06 (1.03 – 1.10)	<0.001
Rheumatic disease	10 075 (6.0)	3 494 (4.4)	1.38 (1.33 – 1.44)	< 0.001	0.97 (0.90 – 1.05)	0.48
Peptic ulcer disease:	2 148 (1.3)	1 014 (1.3)	1.00 (0.93 – 1.08)	0.90	0.94 (0.85 – 1.04)	0.26
Mild liver disease:	1 792 (1.1)	965 (1.2)	0.87 (0.81 – 0.95)	< 0.001	0.98 (0.88 – 1.11)	0.78
Diabetes without complications	31 961 (19.1)	14 549 (18.5)	1.04 (1.02 – 1.07)	< 0.001	0.96 (0.92 – 1.00)	0.03
Diabetes with complications	15 575 (9.3)	6 695 (8.5)	1.11 (1.07 – 1.14)	< 0.001	1.04 (0.99 – 1.10)	0.11
Hemiplegia or paraplegia:	2 236 (1.3)	1 111 (1.4)	0.95 (0.88 – 1.02)	0.15	0.80 (0.73 – 0.89)	<0.001
Renal disease:	10 698 (6.4)	3768 (4.8)	1.36 (1.31 – 1.41)	< 0.001	0.99 (0.93 – 1.04)	0.58
Cancer	20 058 (12.0)	7325 (9.3)	1.33 (1.29 – 1.37)	< 0.001	1.02 (0.95 – 1.10)	0.63
Moderate or severe liver disease	308 (0.2)	180 (0.2)	0.81 (0.67 – 0.97)	0.02	0.93 (0.72 – 1.23)	0.62
Metastatic cancer	3067 (1.8)	1281 (1.6)	1.13 (1.06 – 1.21)	< 0.001	0.85 (0.77 – 0.93)	<0.001
AIDS/HIV	34 (0.0)	24 (0.0)	0.67 (0.40 – 1.14)	0.13	1.01 (0.49 – 2.16)	0.98

*OR* Odds ratio, 95% *CI* 95% Confidence intervals.

^a^Adjusted by immunosuppression, COVID-19 vaccine history, age, sex and previous Covid hospitalization.

^b^Diagnoses in the Charlson Comorbidity index^14^ past two years (yes, no; Reference=No).

In all, 519 COVID-19-related hospitalizations and 4822 all-cause deaths were recorded during the study period. Among vaccinated individuals, 2607 deaths were recorded (10.3 per 100,000 person-days) compared to 2215 deaths in the unvaccinated (15.8 per 100,000 person-days), while COVID-19–related hospitalizations occurred in 224 vaccinated individuals (0.9 per 100,000 person-days) compared to 295 hospitalizations in the unvaccinated (2.1 per 100,000 person-days) (Table 1).

### Vaccine effectiveness

After adjustment for age group, sex, immunosuppression status, Charlson Comorbidity Index findings, and country of birth, the VE against COVID-19 hospitalization in the total population was 75% (95% CI 70%–79%) in the period October 1, 2024 to March 31, 2025 (Table 4). Evaluation of proportional hazards using Schoenfeld residual test showed no significant time dependence of the vaccine effect (*p* = 0.17). Accordingly, the vaccine protection did not differ between the early and late parts of the period (first 90 days: adjusted VE (aVE) 75% [95% CI 69%–79%]; after 90 days: aVE 74% [95% CI 61%-83%]). Neither did the protection differ with immunosuppression status (immunocompetent individuals: aVE 75% [95% CI 68%–80%]); immunocompromised individuals: aVE 76% [95% CI 68%–82%]) (Table 5).

**Table 4:** Effectiveness of updated COVID-19 vaccine on COVID-19 hospitalizations and negative control outcome (all-cause mortality) in the total population and subpopulations

	Vaccine effectiveness (COVID-19 hospitalization)		Negative control outcome (all-cause mortality)	
	Unadjusted *VE* % (95% CI)	Adjusted^a^ *VE* % (95% CI)	Unadjusted HR (95% CI)	Adjusted^a^ HR (95% CI)
**Total**	64 (57 – 70)	75 (70 – 79)	0.62 (0.59 –0.66)	0.43 (0.40 – 0.45)
**Age groups (yrs.)**				
65-69	67 (34 – 84)	70 (39 – 85)	0.42 (0.33 - 0.55)	0.39 (0.30 – 0.51)
70-74	68 (46 – 81)	72 (52 – 84)	0.47 (0.39 - 0.57)	0.44 (0.36 – 0.53)
75-79	76 (65 – 84)	78 (67 – 85)	0.42 (0.36 - 0.49)	0.40 (0.34 – 0.46)
80-84	69 (55 – 78)	71 (59 – 80)	0.39 (0.34 - 0.44)	0.36 (0.31 – 0.41)
85+	73 (63 – 79)	76 (67 – 82)	0.52 (0.48 - 0.57)	0.48 (0.44 – 0.52)
**Sex**				
Female	67 (57 – 74)	75 (68 – 80)	0.65 (0.60 – 0.71)	0.46 (0.43 – 0.50)
Male	61 (51 – 69)	75 (67 – 81)	0.59 (0.55 – 0.65)	0.39 (0.36 – 0.42)
**Immunosuppression**^b^				
Immunocompetent	63 (54 – 70)	75 (68 – 80)	0.68 (0.63 – 0.73)	0.44 (0.41 – 0.48)
Immunocompromised Immunocompromised	71 (61 – 78)	76 (68 – 82)	0.45 (0.41 – 0.50)	0.38 (0.35 – 0.42)
**Country of birth**				
Sweden	67 (61 – 73)	75 (70 – 79)	0.57 (0.53 – 0.60)	0.42 (0.39 – 0.44)
Other	55 (5 – 78)	74 (44 – 88)	1.07 (0.89 – 1.29)	0.58 (0.48 – 0.70)

*VE* Vaccine effectiveness, *HR* Hazard ratio, 95% *CI* 95% confidence interval.

^a^ Adjusted by age group (65-69, 70-74, 75-79, 80-84 and 85 + ), sex, Immunosuppression status, Charlson Comorbidity index and country of birth.

^b^ Diagnoses used for classification as immunocompromised: B20*, B21*, B22*, B23*, C*, D279, D610, D612, D619, D700, D80*, D810, D811, D812, D814, D815, D816, D817, D818, D819, D82*, D83*, D84*, D86*, D890, D891, D893, D894, D898, D899, E851, E852, E853, E854, E858, E859, G35*, J679, K703, K704, K72*, K743, K744, K745, K746, L930, L932, L94*, M05*, M06*, M07*, M08*, M30*, M313, M315, M32*, M33*, M34*, M353, M359, M460, M461, M468, M469, N04*, O987, R180, T860, T861, T862, T863, T864, T865, Z21*, Z482, Z510, Z511, Z94*.

**Table 5:** Vaccine effectiveness and negative control outcomes in groups defined by immunosuppression status, COVID-19 vaccination history and their interactions

	Vaccine effectiveness (COVID-19 hospitalization)		Negative control outcome (all-cause mortality)	
	Unadjusted VE % (95% CI)	Adjusted^a^ VE % (95% CI)	Unadjusted HR (95% CI)	Adjusted^a^ HR (95% CI)
**Immunosuppression**				
Immunocompetent	63 (54 – 70)	75 (68 – 80)	0.68 (0.63 – 0.73)	0.44 (0.41 – 0.48)
Immunocompromised	71 (61 – 78)	76 (68 – 82)	0.45 (0.41 – 0.50)	0.38 (0.35 – 0.42)
**COVID-19 vaccination history**				
Vaccinated previous year	82 (77 – 85)	84 (80 – 87)	0.32 (0.29 – 0.34)	0.27 (0.25 – 0.29)
Basic vaccination (≥2 doses)	59 (21 – 79)	65 (34 – 82)	1.06 (0.89 – 1.27)	0.90 (0.75 – 1.07)
**Immunosuppression by vaccination history**				
Immunocompetent				
Vaccinated previous year	81 (76 – 86)	84 (79 – 88)	0.35 (0.32 – 0.39)	0.29 (0.26 – 0.32)
Basic vaccination (≥2 doses)	67 (19 – 87)	72 (29 – 89)	1.02 (0.81 – 1.28)	0.89 (0.71 – 1.12)
Immunocompromised				
Vaccinated previous year	83 (76 – 88)	84 (78 – 89)	0.25 (0.22 – 0.28)	0.24 (0.21 – 0.26)
Basic vaccination (≥2 doses)	51 (-25 – 81)	57 (-11 – 83)	0.99 (0.75 – 1.30)	0.90 (0.69 – 1.19)

*VE* Vaccine effectiveness, *HR* Hazard ratio, *95%* *CI* 95% confidence interval.

^a^ Adjusted by age group (65-69, 70-74, 75-79, 80-84 and 85 + ), sex, Immunosuppression status, Charlson Comorbidity Index and country of birth. Diagnoses used for classification as immunocompromised: B20*, B21*, B22*, B23*, C*, D279, D610, D612, D619, D700, D80*, D810, D811, D812, D814, D815, D816, D817, D818, D819, D82*, D83*, D84*, D86*, D890, D891, D893, D894, D898, D899, E851, E852, E853, E854, E858, E859, G35*, J679, K703, K704, K72*, K743, K744, K745, K746, L930, L932, L94*, M05*, M06*, M07*, M08*, M30*, M313, M315, M32*, M33*, M34*, M353, M359, M460, M461, M468, M469, N04*, O987, R180, T860, T861, T862, T863, T864, T865, Z21*, Z482, Z510, Z511, Z94*.

Among individuals who had received the Omicron XBB.1.5-adapted vaccine in 2023, the aVE against COVID-19 hospitalizations was higher than in the total population (84% [95% CI 80%–87%]) (Table 5), while it was lower in those who had not received this vaccine (65% [95% CI 34%–82%]). Similar levels of protection were seen irrespective of immunosuppression status.

### Negative control outcome

The risk of the NCO (all-cause mortality) among vaccinated individuals was less than half compared to the individuals not exposed to the study vaccine (adjusted HR (aHR) 0.43 [95% CI 0.40–0.45]) (Table 4). The Schoenfeld residual test indicated violation of the proportional hazard assumption (*p* < 0.01). The difference was larger in the early part of the evaluation period (first 90 days aHR 0.34 (95% CI 0.32–0.37] than in the later part (after 90 days aHR 0.60 [95% CI 0.54-0.65) (Supplementary Fig. S1). Among individuals having received the Omicron XBB.1.5-adapted vaccine in 2023, the NCO was approximately one quarter as likely in those also exposed to the study vaccine in 2024 compared with those who were not exposed to the vaccine (aHR 0.27 [95% CI 0.25–0.29 (Table 5), while no statistically significant difference in NCO by vaccine exposure status in 2024 was seen in individuals who had not received the updated COVID-19 vaccine the previous year (aHR 0.90 [95% CI 0.75–1.07]).

### Healthy vaccinee effect size model

**Fig. 2:**
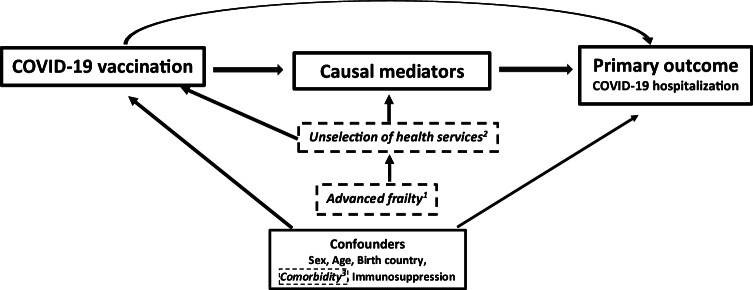
Counterfactual scenario model for quantifying the healthy vaccinee effect. Reduction of the effect is simulated by elimination of causal mediators (marked boxes): advanced frailty^1^ (modeled by removal of individuals factually deceased during the study period)), selective under-provision of preventive measures such as vaccination^2^ (modeled by assuming all individuals diagnosed with metastatic cancer or para/hemiplegia to be vaccinated) and indicated interventions associated with comorbidity^3^ (not correcting for the Charlson Comorbidity Index).

In a reduced healthy vaccinee effect scenario including fewer individuals with end-stage frailty (individuals factually deceased during the study period removed), diminished selective under-provision of vaccination (assuming unvaccinated individuals diagnosed with metastatic cancer and hemi/paraplegia to have been vaccinated), and with interventions indicated by comorbidity cut down (correction for the Charlson Comorbidity Index removed in the analysis) (Fig. 2), the aVE against COVID-19 hospitalization was lower (69% [95% CI 62%–74%]) than factually observed. The counterfactual aVE estimate did not differ significantly between the early and late parts of the evaluation period (first 90 days: 70% [95% CI 63% - 76%]; after 90 days 64% [95% CI 44% - 77%]). When adjustment for comorbidity was introduced to the scenario, the aVE returned to levels comparable to the primary analysis (74% [95% CI 68%–79%]), while it remained consistent over time (first 90 days: 75% [95% CI 69% - 80%]; after 90 days: 70% [95% CI 54% - 81%]).

### NCO validation model

**Fig. 3:**
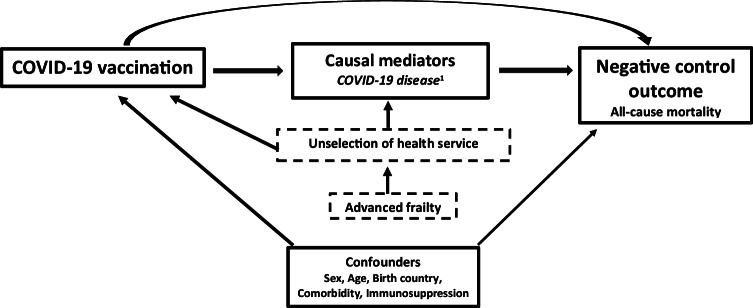
Counterfactual scenario model for comparison of all-cause mortality and non-COVID-19 mortality as negative control outcome in evaluations of updated COVID-19 vaccines in elderly populations. The causal mediator eliminated in the comparison (marked in the box) is COVID-19 disease (COVID-19 hospitalization). Adjustments are made for confounding by sex, age, birth country, comorbidity (Charlson Comorbidity Index), and immunosuppression.

When examining the use of non-COVID-19 mortality as an alternative NCO by removing the 519 individuals diagnosed and hospitalized with COVID-19 from the analysis model (Fig. 3), the association between COVID-19 vaccination and the NCO remained similar to the primary analysis (aHR 0.45 [95% CI 0.42–0.47]). The association also remained larger in the early part of the study period (first 90 days: aHR 0.36, 95% CI 0.34–0.39) than in the later part (after 90 days: aHR 0.56, 95% CI 0.51–0.62).

### Sensitivity analysis

The minimum strength of association on the rate ratio scale that a hypothetical unmeasured confounder would need to have with both vaccination and COVID-19-related hospitalization to explain away the estimated aVE was of large size (lower E-value limit 7.2) (Supplementary Table S1).

## Discussion

In our study involving a total elderly population covering the entire period of Omicron JN.1 lineage circulation in 2024–2025, the observed VE of the BNT162b2 vaccine was found to largely correspond to that reported from the early Danish cohort study^5^. Accordingly, and as in previous studies, no association between immunosuppression status and the effectiveness of the vaccine was found^4^. However, our VE estimate changed markedly when the analysis was restricted according to receipt of the updated COVID-19 vaccine in the previous year. We also observed that the NCO, all-cause mortality, was less than half as frequent in the elderly exposed to vaccination in the study year, compared to those unexposed. These findings suggest our VE estimate was affected by a healthy vaccinee effect.

The aVE in the elderly subpopulation vaccinated with an updated COVID-19 vaccine the previous year was 9% higher than in the total population (84%/75%), while it was 10% lower (65%/75%) in the elderly who had not received the previous vaccine. Similarly, although NCO all-cause mortality was expected to be equally common in the exposed and unexposed groups to the 2024 COVID-19 vaccine during the study period, it was less than half as frequent in the exposed group as in the unexposed group. The difference was larger early in the evaluation period, likely due to fewer individuals with critically poor health remaining in the unexposed subpopulation later in the period. These observations imply that selection effects should be considered when interpreting results from evaluations of updated COVID-19 vaccines in the elderly^12,13^. The observations also suggest that studies requiring prior receipt of an updated COVID-19 vaccine, such as in the recent Danish cohort study^5^, and investigations of humoral and memory B-cell responses to multiple vaccine doses^16,17^, should account for a possible cumulative selection effect.

Considering the magnitude of the healthy vaccinee effect, our analysis of the counterfactual scenario with causal mediators excluded from the model suggested only a modest influence. When we attempted to reduce the effect by excluding individuals with end-stage frailty (those factually deceased during the study period), counter to the fact reduced selective under-provision of preventive intervention (assumed all unvaccinated individuals diagnosed with metastatic cancer or hemi/paraplegia to have been vaccinated), and refrained to correct for indicated health service interventions associated with comorbidity, the VE decreased by 6% (from 75% to 69%), thus remaining substantial. This modest influence was anticipated considering the large size of the E value, but it is still noteworthy, as it highlights disadvantages associated with using medical care utilization as an endpoint in studies of vaccine effectiveness in the elderly.

Accordingly, we do not propose that our findings invalidate the early reports on the effectiveness of the 2024 BNT162b2 vaccine in preventing COVID-19 hospitalization. However, our results raise questions about bias adjustments and endpoint selection in vaccine evaluations among the elderly. In our study setting, no elderly subpopulation was excluded from the recommendation of receiving the updated vaccine. Nonetheless, we observed that a fraction of the elderly with poor health did not receive the updated vaccine to the same extent as their healthier peers. However, the fact that excluding individuals hospitalized for COVID-19 did not affect differences in all-cause mortality between those exposed and unexposed to the study vaccine, and that the VE estimates using COVID-19-related hospitalization as the outcome remained consistent over the study period, suggests that individuals in critically poor health also were less likely to utilize emergency hospital care. This brings to light a latent “phenotype” of elderly with frailty^18^ left unselected for both primary and secondary prevention, i.e. a phenotype associated with underutilization of medical care. This category of frail elderly not receiving health services to the same extent as their healthier peers needs to be identified and considered in the design of vaccine evaluations, as it may affect valid estimations of vaccine effectiveness.

Although we corrected our analyses for confounding by age, sex, birth country, immunosuppression, and comorbidity^6,13,18,19^, major differences in the NCO between the groups exposed and not exposed to the study vaccine showed that these corrections were insufficient to adjust for variations in baseline health. Frailty is an age-related health condition characterized by decreased physiological reserve and increased vulnerability to stressors, predisposing for premature mortality^20^. Data on these reserves and vulnerabilities were not collected in the health care database used in our study and thus could not be used for bias adjustment. This implies that confounding by frailty likely affected our results.

Generalizing from this experience, we find that frailty measures are needed for adequate adjustments for confounding bias in evaluations of VE in the elderly. Frailty can be misunderstood as part of the normal ageing process, causing individuals with frailty to be considered only based on their sociodemographic and clinical characteristics^19,20^. To be able to adjust for the healthy vaccinee effect in vaccine evaluations, measures that adequately represent frailty need to be included in analysis models, particularly if the outcomes of interest include health care utilization.

Moreover, additional research is needed on vaccine recommendations for elderly individuals. Assessment of an elderly individual’s degree of frailty on a spectrum from fit to severely frail provides a framework for applying principles of geriatric care to vaccination practice. Our results show that also the elderly subpopulations with poorer health, for instance, those who had not received the COVID-19 vaccine the previous year (Table 4), still benefitted from the updated vaccine (VE 65%). Heightened vulnerability makes the frail elderly benefit from vaccination, as it prevents avoidable physiological stressors. Incorporating frailty into studies of vaccine effectiveness will promote the formulation of more precise recommendations concerning updated COVID-19 vaccines for elderly populations, also including frail individuals approaching the end of life.

Use of all-cause mortality instead of non-COVID-19 mortality as NCO in an evaluation of COVID-19 vaccines can be questioned. Identification of non-COVID-19 deaths was found challenging at the time of the evaluation because official death certificate data on COVID-19-related hospital mortality had been shown to have poor reliability^21^ and community-level SARS-CoV-2 testing was inconsistent. Nonetheless, exclusion of patients hospitalized with positive SARS-CoV-2 test from the analysis in the counterfactual NCO validation scenario did not change our results. Additionally, we used the CDC definition from the VISION and IVY networks^4^ to correct immunosuppression, whereby about one individual in five was classified as immunocompromised. A narrower and more precise definition of immunosuppression could have been used, but we do not anticipate that changing the definition would meaningfully have influenced our results.

In summary, we found in the total Swedish elderly population a moderately inflated VE of the 2024-2025 COVID-19 vaccine related to a healthy vaccinee effect. This effect was concentrated among a frail subpopulation receiving limited health services. Although reduced, the protection provided by the updated vaccine was still substantial, also in the subpopulation with frailty. Because the elderly and patients with co-morbidities are currently prioritized for COVID-19 vaccination, we advise that the healthy vaccinee effect be routinely assessed in effectiveness evaluations, and that individuals with advanced frailty are addressed in vaccine recommendations.

## Methods

### Ethics statement

The study was approved by the Swedish Ethical Review Authority (EPM 2023-05203-02).

### Study design

Our evaluation of the updated BNT162b2 vaccine used a cohort study design based on the total population aged ≥65 years on October 1, 2024, in Southeast Sweden. No other COVID-19 vaccine was distributed during the study period. The primary evaluation compared COVID-19 hospitalization rates between individuals with and without Omicron JN.1 vaccination across the total population. The healthy vaccinee effect was investigated by analyzing 1) the VE in subpopulations selected by COVID-19 vaccination status in 2023 and 2) the NCO all-cause mortality among individuals who had and had not received the COVID-19 vaccine in 2024. The data for the primary outcome were collected from patients hospitalized at least overnight with the diagnosis COVID-19, virus identified (the International Classification of Diseases, 10^th^ Revision, code U07.1). All patients hospitalized during the study period were given a PCR test for virus identification and diagnosis. Individual-level data for the NCO all-cause mortality were collected from census records. Analyses of counterfactual scenarios were used to estimate the magnitude of healthy vaccinee effects and to validate the NCO.

### Data sources

The evaluation data were retrieved from the digital health platform developed by the Southeast Healthcare Region^22^. The Southeast Healthcare Region (SEHR) is the association of the local-government financed healthcare providers delivering health services to the residents in Östergötland, Jönköping, and Kalmar Län counties in Southeast Sweden (total population 1,200,000). Before the COVID-19 pandemic, SEHR had developed a digital health platform for service quality assessment and population health surveillance. In supporting the management of population health emergencies, the platform included information resources for community surveillance, intervention design, and analyses of outcomes and impacts^23–25^. At the early stages of the COVID-19 pandemic, syndromic data on COVID-19 symptoms recorded at the platform from telenursing consultations were used to forecast local hospital capacity needs^26^.

### Vaccine coverage

We analyzed variations in vaccination coverage using a multivariable logistic regression model with COVID-19 vaccination as the dependent variable and, as independent variables, age group, sex, morbidity in any of the 17 diagnoses included in the updated weighted Charlson Comorbidity Index^27^, immunosuppression status^4^, previous vaccination (yes, no), and country of birth.

### Primary and negative control outcomes

The primary evaluation compared the rates of COVID-19 hospitalization between individuals with and without JN.1 vaccination using a Cox regression model based on time from index date, adjusting for age group (5-year intervals), sex, updated weighted Charlson Comorbidity Index^27^, and immunosuppression status (competent, compromised)^4^. The index date was set to record the first vaccination or October 1, 2024, if not vaccinated during the period October 1, 2024, to March 31 2025. Vaccine effectiveness was calculated by (1-Hazard Ratio (HR))*100.

The healthy vaccinee effect was first investigated by analyzing VE separately in subpopulations selected by COVID-19 vaccination status (yes/no) in 2023. The effect was further analyzed by comparing the NCO all-cause mortality in individuals with and without JN.1 vaccination using a Cox regression, adjusting for age group (5-year intervals), sex, immunosuppression status (competent, compromised), and updated weighted Charlson Comorbidity Index. This outcome was used as an NCO under the assumption that COVID-19 vaccination has no causal effect on short-term all-cause mortality (within six months). Kaplan-Meier plots were produced for VE and NCO and the proportional hazard assumption was assessed by the Schoenfeld residual test. Regression models were applied to the entire study population as well as to subpopulations defined by age, sex, immunosuppression status, country of birth, and vaccination history. Participants were in analyses of COVID-19 hospitalizations followed up until hospitalization (ICD10 code U07.1), death, or the end of the study period (March 31, 2025). In analyses of all-cause mortality, the participants were followed until death or the end of the study (March 31, 2025). To confirm a healthy vaccine effect, separate analyses were performed for the first 90 days of the evaluation period and after 90 days, respectively. All statistical tests were two-sided, and *p*-values less than 0.05 were considered statistically significant. No adjustment for multiple comparisons was applied. All analyses were conducted using R version 4.5.2.

### Counterfactual simulations

Counterfactual scenario models were simulated to quantify the healthy vaccinee effect and validate all-cause mortality as NCO for the evaluation. The scenarios were constructed to study the impact of the elimination of factors mediating the effect of an exposure on an outcome of interest. The first scenario represented the elimination of factors mediating the healthy vaccinee effect: individuals factually deceased during the study period were assumed not to reside in the study county, unvaccinated individuals diagnosed with metastatic cancer and hemi/paraplegia were assumed to be vaccinated, and correction for indicated interventions associated with disorders included in the Charlson index was removed from the adjustments for confounding bias (Fig. 2).

A second counterfactual scenario model was aimed at validating all-cause mortality as NCO for the evaluation. For this purpose, individuals hospitalized with COVID-19 and corrected for the Charlson index were removed from the analysis of differences in all-cause mortality between vaccinated and unvaccinated individuals and the effects on outcome observed and compared with the factual results (Fig. 3).

### Sensitivity analyses

A sensitivity analysis was performed to assess the robustness of the primary exposure–outcome relationships (between COVID-19 vaccination and COVID-19 hospitalization) to unmeasured confounding by calculation of the E-value. The E value quantifies the minimum strength of association on the rate ratio (RR) scale that a hypothetical unmeasured confounder would need to have with both the outcome and the exposure to explain away the estimated exposure–outcome association^28^. The confidence limit of the E value closest to the null is of higher interest than the E value of the point estimate. This confidence limit is calculated by replacing the RR by the lower or the upper confidence limit of the RR, depending on which of the two are closest to the null (if RR > 1, the lower confidence limit is closest to the null; and if RR < 1, the upper confidence limit is closest to the null). Because the outcome measure used in this study is the hazard ratio (HR) rather than the RR, the E value was calculated differently depending on whether the outcome was rare (prevalence approximately < 15% or > 85%) or not rare (prevalence approximately between 15% and 85%).

If the outcome was rare ( < 15%), the HR approximates the RR and the original E value formulas were used (LL = Lower limit, UL = Upper limit):

1 $${If}\,{HR}\, > \,1: 	 E\,{value}\,({Point}\,{estimate})={HR}+\sqrt{{HR}*\left({HR}-1\right)}\\ \,	{E\; value}\left({LL}\right)=1\,{if\; LL}\le 1,\,{else}\,\,{LL}+\sqrt{{LL}*({LL}-1)}.\\ {If}\,{HR}\, < \,1: 	 E\, {value}\,\,({Point}\,{estimate})={1/HR}+\sqrt{{1/HR}*\left({1/HR}-1\right)}\\ \,	{E\; value}\,\,\left({UL}\right)=1\,{if\; UL}\ge 1,\,{else}\,\,1/{UL}*\sqrt{1/{UL}*(1/{UL}-1)}.$$

Else an approximate E value was obtained by replacing the RR with the square root of the HR:

2 $${If}\,{HR}\, > \,1: 	 E\,{value}\,({Point}\,{estimate})=\sqrt{HR}+\sqrt{{HR}*\left({HR}-1\right)}\\ \, 	 E\,{value}\,\,\left({LL}\right)=1\,{if}\,{LL}\le 1,{else}\,\,1/\sqrt{{LL}}+\sqrt{1/\sqrt{{LL}}*\left(1/\sqrt{{LL}}-1\right)}.\\ {If}\,{HR} < 1: 	 E\,{value}\,\,\left({Point}\,{estimate}\right)=1/ \sqrt{{HR}}+\sqrt{1/\sqrt{{HR}}*\left(1/\sqrt{{HR}}-1\right)}\\ \,	 E\,{value}\,\,\left({UL}\right)=1\,{if}\,{UL}\ge 1,\,{else}\,\,1/\sqrt{{UL}}+\sqrt{1/\sqrt{{UL}}*\left(1/\sqrt{{UL}}-1\right)}.$$

Higher E values correspond to situations in which the existence of an unmeasured confounder able to tip the estimated exposure–outcome relationship is more unlikely.

### Reporting summary

Further information on research design is available in the Nature Portfolio Reporting Summary linked to this article.

## Supplementary information

Supplementary Information

Reporting summary

Transparent Peer Review file

## Data Availability

The data used for the study are available under restricted access to preserve the confidentiality of patient information. No datasets used in this study, whether raw or de-identified, can be publicly released by the researchers. Access can be obtained for research purposes through an application to the data owners. The dataset is the property of the health service providers involved and was provided to the researchers through a restricted-access agreement that prohibits sharing the dataset with third parties or making it publicly available. Individuals or entities interested in accessing the data may contact the health service providers by email (forskningsdata@regionostergotland.se). All proposed research must obtain the necessary ethical approvals and fulfill legal requirements for issuance of research data from Swedish administrative health service registers.

## Code availability

The R code used for the analyses is available for inspection and download at Code Ocean (10.24433/CO.2906014.v1).
