# Supplementary material for: Healthy vaccinee effect in the evaluation of updated COVID-19 vaccines in elderly populations
Source: Nat Commun. 2026 Aug 8;17:8014. doi: 10.1038/s41467-026-76312-x (PMC13452808; doi:10.1038/s41467-026-76312-x)
Supplement: Supplementary file 1 [file 41467_2026_76312_MOESM2_ESM.pdf]

nature portfolio

Corresponding author(s): DAPR NCOMMS-25-82556A

Last updated by author(s): Jun 16, 2026

Reporting Summary

Nature Portfolio wishes to improve the reproducibility of the work that we publish. This form provides structure for consistency and transparency in reporting. For further information on Nature Portfolio policies, see our Editorial Policies and the Editorial Policy Checklist.

Statistics

For all statistical analyses, confirm that the following items are present in the figure legend, table legend, main text, or Methods section.

n/a	Confirmed	
☐	☒	The exact sample size (*n*) for each experimental group/condition, given as a discrete number and unit of measurement
☐	☒	A statement on whether measurements were taken from distinct samples or whether the same sample was measured repeatedly
☐	☒	The statistical test(s) used AND whether they are one- or two-sided<br>*Only common tests should be described solely by name; describe more complex techniques in the Methods section.*
☐	☒	A description of all covariates tested
☐	☒	A description of any assumptions or corrections, such as tests of normality and adjustment for multiple comparisons
☐	☒	A full description of the statistical parameters including central tendency (e.g. means) or other basic estimates (e.g. regression coefficient) AND variation (e.g. standard deviation) or associated estimates of uncertainty (e.g. confidence intervals)
☐	☒	For null hypothesis testing, the test statistic (e.g. *F*, *t*, *r*) with confidence intervals, effect sizes, degrees of freedom and *P* value noted<br>*Give P values as exact values whenever suitable.*
☒	☐	For Bayesian analysis, information on the choice of priors and Markov chain Monte Carlo settings
☒	☐	For hierarchical and complex designs, identification of the appropriate level for tests and full reporting of outcomes
☐	☒	Estimates of effect sizes (e.g. Cohen's *d*, Pearson's *r*), indicating how they were calculated

Our web collection on statistics for biologists contains articles on many of the points above.

Software and code

Policy information about availability of computer code

Data collection	Evaluation data were collected from the healthcare providers in Southeast Sweden (Region Jönköping County, Region Östergötland, Region Kalmar) from 1 October 2024 to 31 March 2025.
Data analysis	All statistical analyses were performed using the R software package version 4.5.2. The code can be accessed at Code Ocean (Capsule 8189cc28-bd55-4b87-85bf-5412ef6831a4).

For manuscripts utilizing custom algorithms or software that are central to the research but not yet described in published literature, software must be made available to editors and reviewers. We strongly encourage code deposition in a community repository (e.g. GitHub). See the Nature Portfolio guidelines for submitting code & software for further information.

Data

Policy information about availability of data

All manuscripts must include a data availability statement. This statement should provide the following information, where applicable:

- Accession codes, unique identifiers, or web links for publicly available datasets
- A description of any restrictions on data availability
- For clinical datasets or third party data, please ensure that the statement adheres to our policy

The data used for the study are available under restricted access to preserve the confidentiality of patient information. No datasets used in this study, whether raw

or de-identified, can be publicly released by the researchers. Access can be obtained for research purposes through application to the data owners. The dataset is the property of the health service providers involved and was provided to the researchers through a restricted-access agreement that prohibits sharing the dataset with third parties or making it publicly available. Individuals or entities interested in accessing the data may contact the health service providers by email (forskningsdata@regionostergotland.se). All proposed research must obtain the necessary ethical approvals and fulfill legal requirements for issuance of research data from Swedish administrative health service registers.

Research involving human participants, their data, or biological material

Policy information about studies with human participants or human data. See also policy information about sex, gender (identity/presentation), and sexual orientation and race, ethnicity and racism.

Reporting on sex and gender	The results are adjusted for sex (male, female). Data on gender were not available.
Reporting on race, ethnicity, or other socially relevant groupings	Vaccine effectiveness results are presented adjusted for age group, sex (male, female), and country of birth (Sweden, other). To explore root causes of healthy vaccinee effects, we analyzed and reported variations in vaccination coverage using a multivariable model with COVID-19 vaccination as dependent variable and as independent variables age group, sex (male, female), support dependency (Charlson Comorbidity index), and country of birth (Sweden, other).
Population characteristics	The covariate-relevant population characteristics used in the analyses were age group, sex (male, female), immunosuppression status (see below*), previous vaccination (year before: yes, no; earlier: yes, no), country of birth (Sweden, other country), and morbidity in any of the 17 diagnoses included in the Charlson's comorbidity index in the previous four years.<br>*The diagnoses used for classification of immunosuppression status were chosen to allow comparison with the VISION and IVY Networks (Link-Gelles 2024): B20*, B21*, B22*, B23*, C*, D279, D610, D612, D619, D700, D80*, D810, D811, D812, D814, D815, D816, D817, D818, D819, D82*, D83*, D84*, D86*, D890, D891, D893, D894, D898, D899, E851, E852, E853, E854, E858, E859, G35*, J679, K703, K704, K72*, K743, K744, K745, K746, L930, L932, L94*, M05*, M06*, M07*, M08*, M30*, M313, M315, M32*, M33*, M34*, M353, M359, M460, M461, M468, M469, N04*, O987, R180, T860, T861, T862, T863, T864, T865, Z21*, Z482, Z510, Z511, Z94*.<br><br>Link-Gelles R CS, Webber A, et al. Interim Estimates of 2024–2025 COVID-19 Vaccine Effectiveness Among Adults Aged ≥18 Years — VISION and IVY Networks, September 2024–January 2025. MMWR Morb Mortal Wkly Rep. 74, 73–82 (2025).
Recruitment	This is a total population study.
Ethics oversight	The study was approved by the Swedish Ethical Review Authority (EPM 2023-05203-02).

Note that full information on the approval of the study protocol must also be provided in the manuscript.

Field-specific reporting

Please select the one below that is the best fit for your research. If you are not sure, read the appropriate sections before making your selection.

☒ Life sciences ☐ Behavioural & social sciences ☐ Ecological, evolutionary & environmental sciences

For a reference copy of the document with all sections, see nature.com/documents/nr-reporting-summary-flat.pdf

Life sciences study design

All studies must disclose on these points even when the disclosure is negative.

Sample size	This was a total population study.
Data exclusions	No data exclusions were made.
Replication	No formal replications were performed. Sensitivity analyses were applied to the evaluation data to assess the effects of deleting population factions from the data set (such as individuals diagnosed and hospitalized with COVID-19) and changing their vaccination status. The sensitivity analyses also included assessing the robustness of the main exposure–outcome relationships of interest to unmeasured confounding by calculation of the E-value. The E value quantifies the minimum strength of association on the rate ratio (RR) scale that a hypothetical unmeasured confounder would need to have with both the outcome and the exposure to explain away the estimated exposure–outcome association. The confidence limit of the E value closest to the null was calculated by replacing the RR by the lower or the upper confidence limit of the RR, depending on which of the two are closest to the null (if RR > 1, the lower confidence limit is closest to the null; and if RR < 1, the upper confidence limit was closest to the null). Because the outcome measure used in this study was the hazard ratio (HR) instead of the RR, the E value was calculated differently depending on whether the outcome was rare (prevalence approximately <15% or >85%) or was not rare (prevalence approximately between 15% and 85%).
Randomization	This was an observational study. No randomization was performed. To emulate randomization and reduce bias, the evaluation data were analyzed using Cox proportional hazard models adjusted for age, sex, immunosuppression status, and country of birth. To explore root causes of healthy vaccinee effects, we analyzed variations in vaccination coverage using a multivariable model with COVID-19 vaccination as dependent variable and as independent variables age group, sex, immunosuppression status, previous vaccination (yes, no), country of birth, and morbidity in any of the 17 diagnoses included in the Charlson's comorbidity index in the previous four years.

Blinding	No blimding was performed.

Reporting for specific materials, systems and methods

We require information from authors about some types of materials, experimental systems and methods used in many studies. Here, indicate whether each material, system or method listed is relevant to your study. If you are not sure if a list item applies to your research, read the appropriate section before selecting a response.

Materials & experimental systems

n/a	Involved in the study	
☒	☐	Antibodies
☒	☐	Eukaryotic cell lines
☒	☐	Palaeontology and archaeology
☒	☐	Animals and other organisms
☐	☒	Clinical data
☒	☐	Dual use research of concern
☒	☐	Plants

Methods

n/a	Involved in the study	
☒	☐	ChIP-seq
☒	☐	Flow cytometry
☒	☐	MRI-based neuroimaging

Clinical data

Policy information about clinical studies

All manuscripts should comply with the ICMJE guidelines for publication of clinical research and a completed CONSORT checklist must be included with all submissions.

Clinical trial registration	We used clinical data in this research project, but no clinical trial was conducted.
Study protocol	We have not registered this study as a clinical trial. This was an observational study. No randomization was performed.and we used population-based health care data collected within the health care systems.
Data collection	Data were collected from the healthcare providers in Southeast Sweden (Region Jönköping County, Region Östergötland, Region Kalmar) from 1 October 2024 to 31 March 2025.
Outcomes	The primary outcome was COVID-19 hospitalization and the negative control outcome all-cause mortality. We used Cox regression to analyze these outcomes.

Plants

Seed stocks	N/A
Novel plant genotypes	N/A
Authentication	N/A
