# Supplementary material for: Healthy vaccinee effect in the evaluation of updated COVID-19 vaccines in elderly populations
Source: Nat Commun. 2026 Aug 8;17:8014. doi: 10.1038/s41467-026-76312-x (PMC13452808; doi:10.1038/s41467-026-76312-x)
Supplement: Supplementary file 2 [file 41467_2026_76312_MOESM3_ESM.pdf]

nature portfolio

Peer Review File

Healthy vaccinee effect in the evaluation of updated COVID-19 vaccines in elderly populations

Corresponding Author: Professor Toomas Timpka

This file contains all reviewer reports in order by version, followed by all author rebuttals in order by version.

Version 0:

Reviewer comments:

Reviewer #2

(Remarks to the Author)
The study appropriately uses a large, population-based cohort of elderly individuals (≥65 years) in Sweden to explore whether conventionally-reported VE estimates are biased as a result of the healthy vaccinee effect. The paper addresses an important issue and is clearly presented but I have several concerns as follows.

Clarify the rationale for changing vaccination status in sensitivity analyses (e.g., metastatic cancer, hemiplegia/paraplegia) — this is unconventional and requires more clearly laid out justification. Why weren't more conventional approaches used to mitigate healthy vaccinee bias (e.g., self-controlled designs, negative control outcomes, IPTW).
Provide more detail on how comorbidities were classified and whether misclassification could influence results. All co-morbidities were classified as 'immunosuppression' but there are many way that these factors could influence both outcomes and the likelihood of being vaccinated aside from suppression of the immune response.

The finding that vaccinated individuals had half the all-cause mortality compared to unvaccinated peers strongly suggests a healthy vaccinee effect. However, it should it should be stated explicitly what this and the sensitivity analyses are testing exactly. As it is a series of comparisons and regressions are presented but its not stated clearly what is being tested in each instance.

For example, by removing the individuals that tested positive and were hospitalized with COVID-19, I suppose they are testing whether COVID was a major contributor to all-cause mortality. It's not clear what is being tested in the latter case, where vaccination status was changed.

Relatedly, clarify why all-cause mortality was used as a comparator outcome, and explicitly state that protection against non-COVID mortality is implausible biologically, reinforcing the presence of bias.

The discussion should expand on how these findings might differ in settings with lower vaccination coverage or higher proportions of frail elderly who remain unvaccinated.

(Remarks on code availability)

Reviewer #3

(Remarks to the Author)
Nature Communications manuscript NCOMMS-25-82556-T

Summary: This retrospective cohort study evaluated the VE of the 2024-2025 COVID-19 vaccine in a Swedish population aged 65 years and older. The impact of vaccination on mortality was also assessed. The authors perform several sensitivity

analyses, including changing vaccination status for select high-risk populations to attempt to estimate the 'healthy vaccinee effect'. I have several concerns with this work. Many have to do with including the basic information required to assess the potential bias or completeness of a VE study, including definitions, data sources, and other concerns. I also have concerns with the interpretability of the approach which alters data.

Comments:

-It is not clear how COVID-19 hospitalizations are defined and this should be defined early as it is the main outcome of interest. How is COVID-19 being defined? Is this by ICD code? By positive test? If so, are there criteria for the timing of the test? Are hospitalizations required to be >24 hours? How are you distinguishing whether the hospitalization is 'for COVID-19' rather than 'with COVID-19'? Do these hospitalizations even require any kind of respiratory disease symptoms? There is something concerning about the fact that there are more than 9 times the number of deaths in this study than COVID-19 hospitalizations. What are the procedures in this region for COVID-19 testing? I am not confident that COVID-19 hospitalizations are being captured accurately and I'm not clear on how they are being defined.

-How is death being defined? The only information offered by the authors regarding death is that "official death certificate data were not used to classify COVID-19-related mortality due to poor reliability". In that case, what was used? How was death captured if it occurred outside of a hospital setting?

-It would help the reader to provide some details about 'the digital health platform developed by the Southeast Healthcare Region.' I am not clear if only a small proportion of the regional population is included, if it is only private providers for example, how vaccination is captured, etc. It would be helpful to have at least high-level details to attempt to assess potential bias and/or completeness of data.

-The study dates need to be clearly stated. This is a very basic requirement for reporting a cohort study. In the Methods the authors state that the "…study based on the population aged 65 and older on October 1, 2024 in Southeast Sweden." There is no study data collection end date given. The end date is buried later. It would be helpful to include full study dates in the Abstract as well.

- I may misunderstand the analyses but when the authors remove all individuals who had a COVID-19 hospitalization, the likelihood of all cause mortality did not change. This is very surprising as it is generally accepted that the vaccine prevents death due to prevention of severe COVID-19. This leads back to my concern that the authors are not accurately capturing COVID-19 in this study, due to either data concerns or larger testing practices in the region.

-The authors state that "participants were followed up until COVID-19 hospitalization, death, or the end of the study period." Does this mean to the first event or whichever came latest? If it is first event, then I can see why removing all of those with COVID-19 hospitalizations would not change death analyses, since they are all censored before the chance for death. This should be considered.

-There is a great deal of emphasis placed on the importance of patients with the very specific comorbidities of metastatic cancer and hemiplegia or paraplegia. There are no data to show how many of these patients are in the cohort. Frequencies for each comorbidity would be helpful to assess the impact of these findings.

-I am concerned about the idea of sensitivity analyses where researchers decide to just alter the data. Perhaps this is commonly done but I am less familiar with this approach. The authors demonstrate that VE is equally high in the immunocompromised group as compared with the immunocompetent group. This means the vaccine prevents the outcome even in the high risk populations. When we take a high risk population that is likely to have a higher rate of the outcome and just change the exposure, this does not reflect the biologic reality that the probability of the outcome would change as well. With this same approach, we could just change a subset of the population that does not have the outcome of interest to have the outcome. This would obviously alter the estimates but I'm not sure if any conclusions reached from this are meaningful.

-(Line 123) The estimate is not included in the confidence intervals.

-In general, and in all of the major COVID-19 VE studies I have seen, the populations with the higher comorbidity burdens are actually more likely to be vaccinated. The authors state that "we conclude that healthy vaccinee effects predispose overestimation of vaccine protection…." And that "this should be regularly highlighted…". In general, it's the sicker populations being vaccinated and therefore the opposite is more likely to be true. On this point, in the introduction they state that "the severely ill or otherwise disadvantaged individuals are less likely to be vaccinated" and cite one paper from Qatar. There are very many published studies that show the opposite.

-In Table S1, the ORs are adjusted for immunosuppression status, among other things. The codes for immunocompromised include C* codes, which I believe include the codes for metastatic cancer. Please confirm that the estimate for likelihood for vaccination among those with metastatic cancer is not being adjusted for the same variable.

(Remarks on code availability)
N.A

Reviewer #4

(Remarks to the Author)
In this paper, the authors analyze health data for older adults from southern Sweden during a period when a COVID booster was available. They assess VE against COVID hospitalization and all-cause mortality, and add a series of sensitivity analyses removing hospitalized cases and reassigning vaccination status to certain groups. The article makes a point about the potential for a healthy vaccinee effect.
While there could be evidence for a health vaccinee effect, and I think it is a valuable discussion, I didn't find this article to effectively convey this. The logic driving the sensitivity analyses was not clear, including why certain groups were reassigned or removed. There were also some signals pointing in the other direction (e.g. higher vaccination uptake among immunocompromised, patients with chronic pulmonary disease) that were not discussed. There seemed to be some analyses reported that were missing from the tables. I offer several specific comments.

- Results should be balanced to include conditions associated with both increased and decreased likelihood of vaccination. Vaccinated individuals more likely to have chronic pulmonary disease (adjusted OR 1.06, p<0.001). More likely to be immunocompromised, etc.
- "But when vaccination status for individuals diagnosed with metastatic cancer and hemi/paraplegia was also changed to vaccinated, the different in vaccine protection was reduced by one third." I don't understand this sensitivity analysis. You changed the vaccination status for all of these individuals? Seems extreme. The reasoning for this particular change is not well stated. Why just metastatic cancer and hemi/paraplegia?
- Why not just remove the hospitalized deaths from the sensitivity analysis of all cause death? Why all hospitalized cases? This is also not clear.
- Where can one find the model for the time periods (Oct-Nov, Dec-Jan, Feb-Mar). I don't see these anywhere in the main text or supplement.
- Minor: Figure S1 is unadjusted, yes? Should be clearly labeled as such.
- Minor: Would recommend labeling Figure S1 with proper months, since authors refer to distinct time periods in the text.
- Minor: Table 2 difficult to view with VE scale and HR scale shown together. Difficult for the reader to process.
- Minor: Methods section seems slightly out of order, which results of Schoenfeld test and E-values given before these methods are defined in later paragraphs.
- Would like methods section to define time scale. Presumably calendar time with vaccination status treated as a time-dependent covariate?

(Remarks on code availability)

Version 1:

Reviewer comments:

Reviewer #2

(Remarks to the Author)
All my concerns from the previous review have been adequately addressed.

(Remarks on code availability)

Reviewer #3

(Remarks to the Author)
Comments have been addressed.

(Remarks on code availability)

Reviewer #4

(Remarks to the Author)
In this paper, the authors have made a number of updates. Clarifications have been helpful. Overall, I find the main analysis of differences in all-cause mortality to be worth publishing. I still find the logic for the simulation to be weaker and does not add much to the paper.
Comments:
- Is there a seasonality to all-cause mortality? Necessary to adjust for differences in timing of index date between vaccinated and unvaccinated?
- Clearly define short-term mortality
- Logic of counterfactual simulations still not great, and would merit further explanation in the methods.
- Table 2. I don't understand how these percentages are calculated. They don't add to 100% in either direction.
- Should mention that some conditions, like chronic pulmonary disease, associated with higher vaccination.

(Remarks on code availability)

Open Access This Peer Review File is licensed under a Creative Commons Attribution 4.0 International License, which permits use, sharing, adaptation, distribution and reproduction in any medium or format, as long as you give appropriate credit to the original author(s) and the source, provide a link to the Creative Commons license, and indicate if changes were made.
In cases where reviewers are anonymous, credit should be given to 'Anonymous Referee' and the source.
The images or other third party material in this Peer Review File are included in the article's Creative Commons license, unless indicated otherwise in a credit line to the material. If material is not included in the article's Creative Commons license and your intended use is not permitted by statutory regulation or exceeds the permitted use, you will need to obtain permission directly from the copyright holder.
To view a copy of this license, visit https://creativecommons.org/licenses/by/4.0/

REVIEWER COMMENTS

Reviewer #2 (Remarks to the Author):

The study appropriately uses a large, population-based cohort of elderly individuals (≥65 years) in Sweden to explore whether conventionally-reported VE estimates are biased as a result of the healthy vaccinee effect. The paper addresses an important issue and is clearly presented but I have several concerns as follows.
Authors' response: Thank you for your insightful comments that have helped us to improve the paper.

Clarify the rationale for changing vaccination status in sensitivity analyses (e.g., metastatic cancer, hemiplegia/paraplegia) — this is unconventional and requires more clearly laid out justification. Why weren't more conventional approaches used to mitigate healthy vaccinee bias (e.g., self-controlled designs, negative control outcomes, IPTW).
Authors' response: We used recent COVID-19 vaccination history (vaccination in the previous year, 2023-2024, with a COVID-19 vaccine adapted to the Omicron XBB.1.5 lineage) and the negative control outcome (NCO) all-cause mortality to determine the presence of a healthy vaccinee effect, as proposed from previous research on vaccine effectiveness among the elderly (Nelson et al. 2009, p. 691). As explained in the revised paper, the healthy vaccinee effect is hard to quantify in elderly populations because frail elderly with critically poor health are difficult to identify in current health service databases. We therefore used recent vaccination history and the NCO to identify the presence of a healthy vaccinee effect and counterfactual simulations, where assumed causes of the effect were eliminated, to quantify the effect. In the latter simulations, individuals factually deceased during the study period were assumed to not reside in the study county, unvaccinated individuals diagnosed with metastatic cancer and hemi/paraplegia assumed to be vaccinated, and corrections for indicated health service interventions removed; see Figure 2.

Reference: Nelson JC, Jackson ML, Weiss NS, Jackson LA. New strategies are needed to improve the accuracy of influenza vaccine effectiveness estimates among seniors. J Clin Epidemiol. 2009 Jul;62(7):687-94. doi: 10.1016/j.jclinepi.2008.06.014.

Provide more detail on how comorbidities were classified and whether misclassification could influence results. All co-morbidities were classified as 'immunosuppression' but there are many way that these factors could influence both outcomes and the likelihood of being vaccinated aside from suppression of the immune response.
Authors' response: We used the classification of immunosuppression employed by Link-Gelles et (2025) in their early evaluation of the 2025-25 COVID-19 vaccine in immunocompromised patients ≥ 65 years of age. We agree that the factors included can be involved in both indication and healthy vaccinee effects. However, while bias due to the indication effect can be associated with relatively homogeneous diagnosis groups and thus analyses corrected through adjustment for the corresponding diagnoses, the same corrections for confounding bias are not readily available for the healthy vaccinee effect. We explain in the revised manuscript that variation in frailty is a likely reason for the healthy vaccinee effect observed in this study and that correction for this factor would require collection of more data. We also comment that more research is needed on classification of immunosuppression.

Reference: Link-Gelles R CS, Webber A, et al. Interim Estimates of 2024–2025 COVID-19 Vaccine Effectiveness Among Adults Aged ≥18 Years — VISION and IVY Networks, September 2024–January 2025. MMWR Morb Mortal Wkly Rep. 74, 73–82 (2025) doi:http://dx.doi.org/10.15585/mmwr.mm7406a1

The finding that vaccinated individuals had half the all-cause mortality compared to unvaccinated peers strongly suggests a healthy vaccinee effect. However, it should it should be stated explicitly what this and the sensitivity analyses are testing more exactly. As it is a series of comparisons and regressions are presented but its not stated clearly what is being tested in each instance.
Authors' response: We explain in the revised paper the rationale(s) behind all analyses (also using graphical models) and what they are testing more exactly. We clarify that the presence of a healthy vaccinee effect was determined through analyses of the COVID-19 vaccine reception status the

previous year and the NCO all-cause mortality. As stated above, the healthy vaccinee effect is hard to quantify because the elderly with poor health likely causing the effect are difficult to identify using current health care databases.

For example, by removing the individuals that tested positive and were hospitalized with COVID-19, I suppose they are testing whether COVID was a major contributor to all-cause mortality. It's not clear what is being tested in the latter case, where vaccination status was changed.
Authors' response: We explain in the revised paper that we aimed to confirm the validity of using all-cause mortality as NCO in a study of updated COVID-19 vaccines by comparing the use of all-cause mortality with non-COVID-19 mortality.

Relatedly, clarify why all-cause mortality was used as a comparator outcome, and explicitly state that protection against non-COVID mortality is implausible biologically, reinforcing the presence of bias.
Authors' response: The revision has been made, including a discussion of that protection against non-COVID mortality is implausible biologically in the Discussion section.

The discussion should expand on how these findings might differ in settings with lower vaccination coverage or higher proportions of frail elderly who remain unvaccinated.
Authors' response: We have expanded the discussion as suggested by the reviewer and also added references.

Reviewer #3 (Remarks to the Author):

Nature Communications manuscript NCOMMS-25-82556-T

Summary: This retrospective cohort study evaluated the VE of the 2024-2025 COVID-19 vaccine in a Swedish population aged 65 years and older. The impact of vaccination on mortality was also assessed. The authors perform several sensitivity analyses, including changing vaccination status for select high-risk populations to attempt to estimate the 'healthy vaccinee effect'. I have several concerns with this work. Many have to do with including the basic information required to assess the potential bias or completeness of a VE study, including definitions, data sources, and other concerns. I also have concerns with the interpretability of the approach which alters data.
Authors' response: Thank you for your time and detailed comments that helped us to significantly improve the paper.

Comments:

-It is not clear how COVID-19 hospitalizations are defined and this should be defined early as it is the main outcome of interest.
Authors' response: We explain in the revised manuscript in detail how COVID-19 hospitalizations were defined (see the details below).

How is COVID-19 being defined? Is this by ICD code? By positive test? If so, are there criteria for the timing of the test?
Authors' response: COVID-19 was defined by the ICD code U07.1 (COVID-19, virus identified), which in turn required a positive SARS-CoV-2 test.

Are hospitalizations required to be >24 hours?
Authors' response: Hospitalization was defined to include hospital admission and over-night stay.

How are you distinguishing whether the hospitalization is 'for COVID-19' rather than 'with COVID-19'? Do these hospitalizations even require any kind of respiratory disease symptoms? There is something concerning about the fact that there are more than 9 times the number of deaths in this study than COVID-19 hospitalizations.
Authors' response: We included all hospitalizations, being aware that that the distinction between 'for COVID-19' rather than 'with COVID-19' is challenging. We have addressed this fact in a separate paper (Spreco et al 2022) and refer to the paper in the discussion.

Reference: Spreco A, Andersson C, Sjödahl R, Dahlström Ö, Timpka T. Hospital bed occupancy for COVID-19 or with SARS-CoV-2 infection in a Swedish county during the omicron wave. Influenza Other Respir Viruses. 2022 Nov;16(6):1194-1196. doi: 10.1111/irv.13019.

What are the procedures in this region for COVID-19 testing? I am not confident that COVID-19 hospitalizations are being captured accurately and I'm not clear on how they are being defined.
<u>Authors' response</u>: All patients admitted to hospital with indication of possible COVID-19 (symptoms or signs) were provided a PCR test for SARS-CoV-2.

-How is death being defined? The only information offered by the authors regarding death is that "official death certificate data were not used to classify COVID-19-related mortality due to poor reliability". In that case, what was used? How was death captured if it occurred outside of a hospital setting?
<u>Authors' response</u>: Information on deaths were routinely added to the database from census records. Cause-of-death data were not used to differ between non-COVID-19 mortality and all-cause mortality due to validity concerns. We discuss the validity of death certificates and have added a reference to a study where we examined the validity of the certificates (Spreco 2023).

Reference: Spreco A, Andersson C, Sjödahl R, Timpka T. Concordance between COVID-19 mortality statistics derived from clinical audit and death certificates in Östergötland county, Sweden. Public Health. Aug;221:46-49.(2023) doi: 10.1016/j.puhe.2023.06.007

-It would help the reader to provide some details about 'the digital health platform developed by the Southeast Healthcare Region.' I am not clear if only a small proportion of the regional population is included, if it is only private providers for example, how vaccination is captured, etc. It would be helpful to have at least high-level details to attempt to assess potential bias and/or completeness of data.
<u>Authors' response</u>: All health care provision is covered by the digital health platform. Information and several references have been added.

-The study dates need to be clearly stated. This is a very basic requirement for reporting a cohort study. In the Methods the authors state that the "…study based on the population aged 65 and older on October 1, 2024 in Southeast Sweden." There is no study data collection end date given. The end date is buried later. It would be helpful to include full study dates in the Abstract as well.
<u>Authors' response</u>: The study dates have been added as suggested.

- I may misunderstand the analyses but when the authors remove all individuals who had a COVID-19 hospitalization, the likelihood of all cause mortality did not change. This is very surprising as it is generally accepted that the vaccine prevents death due to prevention of severe COVID-19. This leads back to my concern that the authors are not accurately capturing COVID-19 in this study, due to either data concerns or larger testing practices in the region.
<u>Authors' response</u>: We explain in the revised paper that we wanted to study the validity of using all-cause mortality as NCO by comparing non-COVID-19 mortality (hospitalized patients with positive SARS-CoV-2 test) and all-cause mortality, respectively. Therefore, a simulation was performed with COVID-19 hospitalization removed.

-The authors state that "participants were followed up until COVID-19 hospitalization, death, or the end of the study period." Does this mean to the first event or whichever came latest? If it is first event, then I can see why removing all of those with COVID-19 hospitalizations would not change death analyses, since they are all censored before the chance for death. This should be considered.
<u>Authors' response</u>: Right-censoring for COVID-19 hospitalization was not performed in the analysis of all-cause mortality. This fact is clarified in the revised paper.

-There is a great deal of emphasis placed on the importance of patients with the very specific comorbidities of metastatic cancer and hemiplegia or paraplegia. There are no data to show how many of these patients are in the cohort. Frequencies for each comorbidity would be helpful to assess the impact of these findings.
<u>Authors' response</u>: Information on frequencies of all comorbidities have been added as suggested.

-I am concerned about the idea of sensitivity analyses where researchers decide to just alter the data. Perhaps this is commonly done but I am less familiar with this approach. The authors demonstrate that

VE is equally high in the immunocompromised group as compared with the immunocompetent group. This means the vaccine prevents the outcome even in the high risk populations. When we take a high risk population that is likely to have a higher rate of the outcome and just change the exposure, this does not reflect the biologic reality that the probability of the outcome would change as well. With this same approach, we could just change a subset of the population that does not have the outcome of interest to have the outcome. This would obviously alter the estimates but I'm not sure if any conclusions reached from this are meaningful.
Authors' response: We used recent COVID-19 vaccination history (vaccination in the previous year, 2023, with a COVID-19 vaccine adapted to the Omicron XBB.1.5 lineage) and the negative control outcome (NCO) all-cause mortality to determine the presence of a healthy vaccinee effect, as proposed from previous research on vaccine effectiveness among the elderly (Nelson et al. 2009, p. 691). As explained in the revised paper, the healthy vaccinee effect is hard to quantify in elderly populations because frail elderly with critically poor health are difficult to identify in current health service databases. We therefore used recent vaccination history and the NCO to identify the presence of a healthy vaccinee effect and counterfactual simulations, where assumed causes of the effect were eliminated, to quantify the effect. In the latter simulations, individuals factually deceased during the study period were assumed to not reside in the study county, unvaccinated individuals diagnosed with metastatic cancer and hemi/paraplegia assumed to be vaccinated, and corrections for indicated health service interventions removed; see Figure 2.

Reference: Nelson JC, Jackson ML, Weiss NS, Jackson LA. New strategies are needed to improve the accuracy of influenza vaccine effectiveness estimates among seniors. J Clin Epidemiol. 2009 Jul;62(7):687-94. doi: 10.1016/j.jclinepi.2008.06.014.

-(Line 123) The estimate is not included in the confidence intervals.
Authors' response: A typo has been corrected.

-In general, and in all of the major COVID-19 VE studies I have seen, the populations with the higher comorbidity burdens are actually more likely to be vaccinated. The authors state that "we conclude that healthy vaccinee effects predispose overestimation of vaccine protection…." And that "this should be regularly highlighted…". In general, it's the sicker populations being vaccinated and therefore the opposite is more likely to be true. On this point, in the introduction they state that "the severely ill or otherwise disadvantaged individuals are less likely to be vaccinated" and cite one paper from Qatar. There are very many published studies that show the opposite.
Authors' response: We emphasize in the revised paper that we address VE in the elderly. In this age group, the healthy vaccinee effect is recognized to dominate (Nelson et al 2009).

Reference: Nelson JC, Jackson ML, Weiss NS, Jackson LA. New strategies are needed to improve the accuracy of influenza vaccine effectiveness estimates among seniors. J Clin Epidemiol. 2009 Jul;62(7):687-94. doi: 10.1016/j.jclinepi.2008.06.014.

-In Table S1, the ORs are adjusted for immunosuppression status, among other things. The codes for immunocompromised include C* codes, which I believe include the codes for metastatic cancer. Please confirm that the estimate for likelihood for vaccination among those with metastatic cancer is not being adjusted for the same variable.
Authors' response: We confirm that metastatic cancer was not adjusted for.

Reviewer #4 (Remarks to the Author):

In this paper, the authors analyze health data for older adults from southern Sweden during a period when a COVID booster was available. They assess VE against COVID hospitalization and all-cause mortality, and add a series of sensitivity analyses removing hospitalized cases and reassigning vaccination status to certain groups. The article makes a point about the potential for a healthy vaccinee effect.

While there could be evidence for a health vaccinee effect, and I think it is a valuable discussion, I didn't find this article to effectively convey this. The logic driving the sensitivity analyses was not clear, including why certain groups were reassigned or removed. There were also some signals pointing in the other direction (e.g. higher vaccination uptake among immunocompromised, patients with chronic pulmonary disease) that were not discussed. There seemed to be some analyses reported that were

missing from the tables. I offer several specific comments.
<u>Authors' response:</u> Thank you for your insightful comments that have helped us to improve the paper. We clarify that the healthy vaccinee effect was studied through analyses of vaccination status in the corresponding program the previous year, through a negative control outcome, and through a counterfactual scenario simulation.

We used recent COVID-19 vaccination history (vaccination in the previous year, 2023-2024, with a COVID-19 vaccine adapted to the Omicron XBB.1.5 lineage) and the negative control outcome (NCO) all-cause mortality to determine the presence of a healthy vaccinee effect, as proposed from previous research on vaccine effectiveness among the elderly (Nelson et al. 2009, p. 691). As explained in the revised paper, the healthy vaccinee effect is hard to quantify in elderly populations because frail elderly with critically poor health are difficult to identify in current health service databases. We therefore used recent vaccination history and the NCO to identify the presence of a healthy vaccinee effect and counterfactual simulations, where assumed causes of the effect were eliminated, to quantify the effect. In the latter simulations, individuals factually deceased during the study period were assumed to not reside in the study county, unvaccinated individuals diagnosed with metastatic cancer and hemi/paraplegia assumed to be vaccinated, and corrections for indicated health service interventions removed; see Figure 2.

Reference: Nelson JC, Jackson ML, Weiss NS, Jackson LA. New strategies are needed to improve the accuracy of influenza vaccine effectiveness estimates among seniors. J Clin Epidemiol. 2009 Jul;62(7):687-94. doi: 10.1016/j.jclinepi.2008.06.014.

- Results should be balanced to include conditions associated with both increased and decreased likelihood of vaccination. Vaccinated individuals more likely to have (adjusted OR 1.06, $p<0.001$). More likely to be immunocompromised, etc.
<u>Authors' response</u>: We agree that the included factors can be involved in both indication and healthy vaccinee effects. Correction for the observed indication effect associated with chronic pulmonary disease did not change the estimate of VE. While the indication effects were relatively straightforward to correct in analyses through adjustment for diagnosed diseases (Charlson index, immunocompromised status, etc.), the same corrections were not readily available for the healthy vaccinee effect. We explain in the revised manuscript that in analysis of VE in the elderly, variability regarding frailty is a likely reason for the effect and that correction for this factor would require recording of more data in health service databases.

- "But when vaccination status for individuals diagnosed with metastatic cancer and hemi/paraplegia was also changed to vaccinated, the different in vaccine protection was reduced by one third." I don't understand this sensitivity analysis. You changed the vaccination status for all of these individuals? Seems extreme. The reasoning for this particular change is not well stated. Why just metastatic cancer and hemi/paraplegia?
<u>Authors' response</u>: As explained in the revised paper, the healthy vaccinee effect is hard to quantify because the unvaccinated group with poor health is difficult to define due to lack of data in health care records. As stated above, we therefore used counterfactual causal mediation analyses, where individuals factually deceased during the study period were assumed to not reside in the study county, unvaccinated individuals diagnosed with metastatic cancer and hemi/paraplegia assumed to be vaccinated, and correction for indicated interventions associated with disorders included in the Charlson index removed from the adjustments for confounding bias to estimate the size of the effect (see Figure 2).

- Why not just remove the hospitalized deaths from the sensitivity analysis of all cause death? Why all hospitalized cases? This is also not clear.
<u>Authors' response</u>: We explain in the revised paper that we aimed to study the validity of using all-cause mortality as NCO by comparing the non-COVID-19 mortality (all individuals hospitalized with positive COVID-19 test excluded) and all-cause mortality, respectively, in vaccinated and unvaccinated individuals. Non-COVID-19 mortality at the population level was not used as NCO due to low reliability of death certificates and low level of SARS-CoV-2 testing in the community, which we have addressed in a previous paper (Spreco et al 2023).

Reference: Spreco A, Andersson C, Sjödahl R, Timpka T. Concordance between COVID-19 mortality statistics derived from clinical audit and death certificates in Östergötland county, Sweden. Public

Health. Aug;221:46-49.(2023) doi: 10.1016/j.puhe.2023.06.007

- Where can one find the model for the time periods (Oct-Nov, Dec-Jan, Feb-Mar). I don't see these anywhere in the main text or supplement.
Authors' response: We added the model to the Methods section.

- Minor: Figure S1 is unadjusted, yes? Should be clearly labeled as such.
Authors' response: The change has been made.

- Minor: Would recommend labeling Figure S1 with proper months, since authors refer to distinct time periods in the text.
Authors' response: Based on the analysis method used, we prefer to keep the study timeline. Because the large majority of vaccinations were performed in October/early November, the difference with calendar time is small.

- Minor: Table 2 difficult to view with VE scale and HR scale shown together. Difficult for the reader to process.
Authors' response: We have considered alternative (more space-consuming) table designs and changing the measures reported (may be confusing) and found problems with both. Therefore, we prefer to keep the table design.

- Minor: Methods section seems slightly out of order, which results of Schoenfeld test and E-values given before these methods are defined in later paragraphs.
Authors' response: The change has been made.

- Would like methods section to define time scale. Presumably calendar time with vaccination status treated as a time-dependent covariate?
Authors' response: We have defined the time scale in the revised manuscript.

RESPONSES TO REVIEWERS' COMMENTS

Reviewer #2 (Remarks to the Author):
All my concerns from the previous review have been adequately addressed.
Authors' response: Thank you for your constructive comments.

Reviewer #3 (Remarks to the Author):
Comments have been addressed.
Authors' response: Thank you for your constructive comments.

Reviewer #4 (Remarks to the Author):
In this paper, the authors have made a number of updates. Clarifications have been helpful. Overall, I find the main analysis of differences in all-cause mortality to be worth publishing. I still find the logic for the simulation to be weaker and does not add much to the paper.
Comments:
- Is there a seasonality to all-cause mortality? Necessary to adjust for differences in timing of index date between vaccinated and unvaccinated?
Authors' response: There is in Sweden some minor seasonality in all-cause mortality (more deaths in winter/summer seasons than in fall/spring). This fact should not have had any meaningful impact on the results of the analysis.

- Clearly define short-term mortality
Authors' response: We have revised the Methods section to explain that short-term mortality was defined as mortality within six months.

- Logic of counterfactual simulations still not great, and would merit further explanation in the methods.
Authors' response: We appreciate this comment and have reviewed the explanation in the Methods section, the legends to Figures 2 and 3, and the figures themselves. Taken together, these elements provide a meaningful explanation of the logic underlying the counterfactual simulations. Nonetheless, if requested, we would be willing to add further clarification.

- Table 2. I don't understand how these percentages are calculated. They don't add to 100% in either direction.
Authors' response: Thank you. Table 2 has been revised to correct a minor error. The percentages do not always add to 100% because of rounding.

- Should mention that some conditions, like chronic pulmonary disease, associated with higher vaccination.
Authors' response: We mention in the Methods, Results, and Discussion that vaccination was indicated for some conditions, including chronic pulmonary disease, and adjusted our analyses accordingly.
