# Supplementary material for: Healthy vaccinee effect in the evaluation of updated COVID-19 vaccines in elderly populations
Source: Nat Commun. 2026 Aug 8;17:8014. doi: 10.1038/s41467-026-76312-x (PMC13452808; doi:10.1038/s41467-026-76312-x)
Supplement: Supplementary file 3 [file 41467_2026_76312_MOESM1_ESM.docx]

**Supplementary information**

Page 1	**Contents**

Page 2	**Figure S1.** Cumulative incidence of death

Page 3	**Table S1** E-values.

**Figure S1.** Cumulative incidence of death in unvaccinated individuals (red) and those vaccinated (blue) with an updated COVID-19 (booster) vaccine in the population aged ≥65 years in the Southeast region of Sweden, October 1, 2024 to March 31, 2025.

**Supplementary table 1.** E values corresponding to the hazard ratios of COVID-19 hospitalizations and negative control outcome (all-cause mortality) in the total population and subpopulations.

|  | **COVID-19 hospitalization** | | **All-cause mortality** | |
| --- | --- | --- | --- | --- |
|  | **Unadjusted E (Lower limit)** | **Adjusted^a^ E (Lower limit)** | **Unadjusted E (Lower limit)** | **Adjusted^a^  E (Lower limit)** |
| **Total** | 5.0 (4.0) | 7.5 (6.1) | 2.6 (2.4) | 4.1 (3.9) |
| **Age groups (yrs.)** | |  |  |  |
| 65-69 | 5.5 (2.4) | 6.1 (2.7) | 4.2 (3.0) | 4.6 (3.3) |
| 70-74 | 5.7 (3.1) | 6.6 (3.6) | 3.7 (2.9) | 4.0 (3.2) |
| 75-79 | 7.8 (5.2) | 8.6 (5.5) | 4.2 (3.5) | 4.4 (3.8) |
| 80-84 | 5.9 (3.9) | 6.4 (4.3) | 4.6 (4.0) | 5.0 (4.3) |
| 85+ | 6.9 (4.8) | 7.8 (5.5) | 3.3 (2.9) | 3.6 (3.3) |
| **Sex** |  |  |  |  |
| Female | 5.5 (4.1) | 7.5 (5.7) | 2.4 (2.2) | 3.8 (3.4) |
| Male | 4.6 (3.5) | 7.5 (5.5) | 2.8 (2.4) | 4.6 (4.2) |
| **Immunosuppression^b^** | |  |  |  |
| Immunocompetent | 4.8 (3.8) | 7.5 (5.7) | 2.3 (2.1) | 4.0 (3.6) |
| Immunocompromised | 6.4 (4.6) | 7.8 (5.7) | 3.9 (3.4) | 4.7 (4.2) |
| **Country of birth** |  |  |  |  |
| Sweden | 5.5 (4.6) | 7.5 (6.1) | 2.9 (2.7) | 4.2 (4.0) |
| Other | 3.9 (1.3) | 7.2 (3.1) | 1.3 (1.0) | 2.8 (2.2) |

E: E value, Lower limit: Lower limit of the 95% confidence interval

^a^ Adjusted by age group (65-69, 70-74, 75-79, 80-84 and 85+), sex, Immunosuppression status, Charlson Comorbidity index and country of birth.

^b^ Diagnoses used for classification as immunocompromised: B20*, B21*, B22*, B23*, C*, D279, D610, D612, D619, D700, D80*, D810, D811, D812, D814, D815, D816, D817, D818, D819, D82*, D83*, D84*, D86*, D890, D891, D893, D894, D898, D899, E851, E852, E853, E854, E858, E859, G35*, J679, K703, K704, K72*, K743, K744, K745, K746, L930, L932, L94*, M05*, M06*, M07*, M08*, M30*, M313, M315, M32*, M33*, M34*, M353, M359, M460, M461, M468, M469, N04*, O987, R180, T860, T861, T862, T863, T864, T865, Z21*, Z482, Z510, Z511, Z94*
